# Depression Associated With Body Mass Index in Adolescent Girls in a Subset of Karachi Population

**DOI:** 10.7759/cureus.24730

**Published:** 2022-05-04

**Authors:** Syeda Sarah Naz, Farah Ahmad

**Affiliations:** 1 Healthcare Management, College of Physician and Surgeons, Karachi, PAK; 2 Community Health Sciences, Ziauddin University, Karachi, PAK

**Keywords:** karachi, adults, socioeconomic status, bmi, depression

## Abstract

Introduction: The prevalence of depression is increasing day by day, which predisposes individuals toward significant functional impairment, and increases the risk of suicide and comorbid physical health problems. Body mass index (BMI) and depression are supposed to be associated with each other; however, the effects of depression on body image have not been identified from the perspective of socioeconomic status, which has been considered a major risk factor for the development of depression. Hence, the study was designed to evaluate the prevalence of depression among adults in government schools and to analyze its association with BMI among different socioeconomic statuses.

Methodology: It was a cross-sectional study conducted at two government schools in Shah Faisal colony from September to October 2019. The study participants were girls of age between 11 and 18 years belonging to different socioeconomic statuses, i.e. low, middle, and high. The calculated sample size was 550 which was calculated at 50% proportion of the total population. A self-developed proforma was administered for collecting demographic data, and students’ weight and height were noted for calculating BMI. The Patient Health Questionnaire (PHQ) 9 modified depression scale was used to assess the depression among study participants. The chi-square test was applied to check the association between BMI and depression score. The study was approved by the IRB of CPSP Karachi and the reference code ME/HCSM/2019/TWC/G-054 was allotted.

Results: There were 345 (62.7%) participants of age 13-15 years, and most of the participants belonged to middle socioeconomic status, 413 (75%). BMI calculation of study participants showed that 417 (75.8%) scored as underweight and 131 (23.8%) had a normal index. According to the PHQ9 scale, 381 (69.3%) participants were having mild depression and 60 (10.9%) had moderate depression. BMI and depression were not associated significantly with a p-value =0.135.

Conclusion: The BMI score of study participants seemed to be underweight or normal. The study could not rule out the association of BMI with depression. However, according to the PHQ9 scale score, many participants screened as sufferers of mild to moderate depression, which is alarming, as depression at the age of 11-18 years may predispose young girls to chronic disease and other psychological conditions.

## Introduction

The prevalence of depression is increasing day by day, which predisposes individuals toward significant functional impairment and increases the risk of suicide and comorbid physical health problems [1]. From 2013 to 2016, 8.1% of adults in the USA suffered from depression; among them, females were affected twice as that as males. Furthermore, National Health and Nutrition Examination Survey revealed that socioeconomic status (SES) was one of the major risk factors in the development of depression that prejudices adults in having difficulty in work, home, and social activities [2,3]. It is documented that residents of low and middle socioeconomic countries experience depression more than that of developed countries and it is becoming a leading cause of disability across the globe [4].

Body mass index (BMI) and depression are supposed to be associated with each other; however, the effects of depression on body image have not been identified yet [5]. Few schools of thought have correlated depression with increased BMI by investigating emotional eating but documented data have not supported the idea as only emotional eating cannot be the reason for an increase in BMI [6]. The influence of increased BMI on depression was further inspected by investigating the role of genes as a predisposing factor and it was found that increased BMI has an association with depression [7]. 

The influence of SES on depression has been correlated negatively, i.e. high-income population suffers less depression when compared to those with low SES [8]. In different studies conducted in Pakistan, depression seemed to be prevalent in university students, predominantly in females, and also it affected the performance grades of students [9,10]. The prevalence of depression in school-going children in India was high and the determined peak age was 18 years [11]. Individuals who belong to low or middle SES can not afford the fee structure of private schools; hence, they prefer government schools to educate their children. The current study was conducted to evaluate the prevalence of depression among adults in government schools and to analyze its association with BMI.

## Materials and methods

Study design and setting

It was a cross-sectional study conducted at two government schools in Shah Faisal colony from September to October 2019.

Inclusion and exclusion criteria

The study participants were girls of age between 11 and 18 years belonging to different SESs, i.e. low, middle, and high. Girls with known depression, psychological conditions, and other systemic disorders (diabetes, hypertension) were excluded.

Sample size and sampling technique

The calculated sample size was 550, which was calculated at 50% proportion of the total population by open-epi software (https://www.openepi.com/SampleSize/SSCohort.htm). The study participants were recruited by convenient sampling. Government schools were approached through proper channels. Before data collection, study objectives were discussed with the recruited participants and written consent for participation was taken.

Study tool

A self-developed proforma was administered for collecting demographic data, and students’ weight and height were noted for calculating BMI. The Patient Health Questionnaire (PHQ) 9 modified depression scale [12] was used to assess the depression among study participants.

Statistical analysis

SPSS v.23 (IBM Corp, Armonk, NY) was used for data analysis, Chi-square test was applied to check the association between BMI and depression score. A p-value less than 0.05% was considered significant. 

Ethical approval

The institutional approval of the study was taken from CPSP Karachi and Reference No.: DME/HCSM/2019/TWC/G-054 was allotted.

## Results

The total calculated sample size was 550 in our study, there were 345 (62.7%) participants of age 13-15 years, and most of the participants belonged to middle SES, 413 (75%). When the participants were asked about feelings of depression, 133 (29.18%) were marked as yes while on the scale the results were different. Table 1 shows the demographic data of study participants.

**Table 1:** Demographic data of study participants

	Frequency	Percentage
Age group		
10-12 years	72	13.2
13-15 years	345	62.7
16-18 years	132	24
Socioeconomic status		
High	31	5.6
Middle	413	75
Low	106	19.2
Parental relationship history		
Happy	543	98.72
Died	7	1.28
History of weight gain		
Yes	96	17.45
No	153	27.8
Don’t know	301	54.7
Psychiatric disease in family		
Yes	27	4.90
No	298	54.18
Don’t know	225	40.90
Feeling insecurity in home		
Father	51	9.27
Brother	63	11.45
Uncle	124	22.5
Nobody	490	89
Feeling of depression		
Yes	133	29.18
No	417	75.8

**Figure 1:**
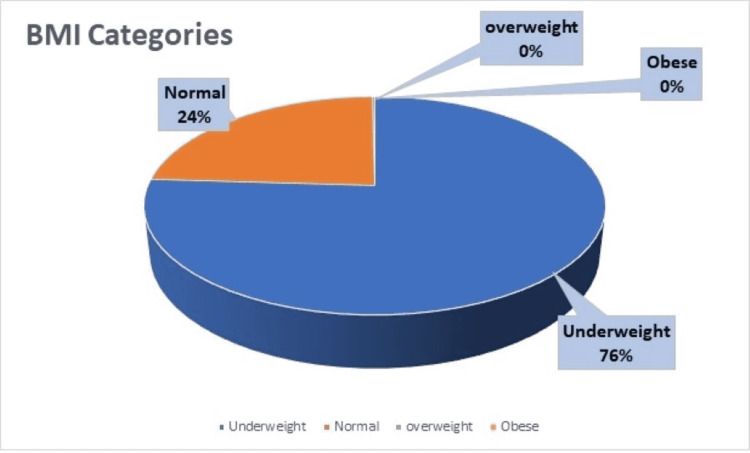
BMI categories of study participants BMI, body mass index.

**Figure 2:**
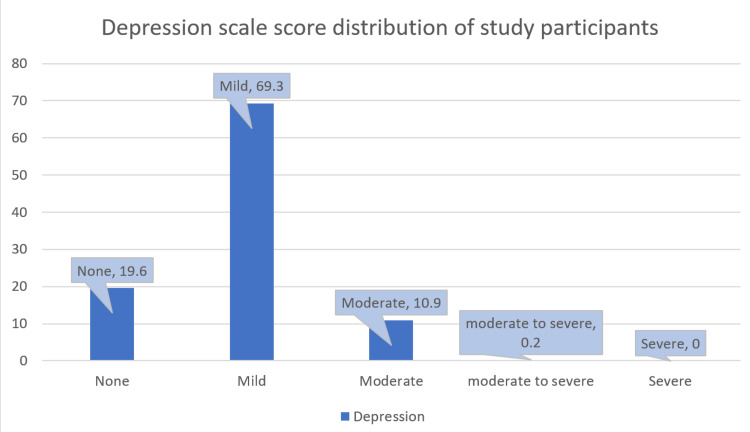
Distribution of participants according to depression score scale

BMI calculation of study participants showed that 417 (75.8%) scored as underweight and 131 (23.8%) had a normal index, which highlighted that the study participants were having normal BMI. Figure 1 shows the BMI of study participants. The depression was analyzed by using the PHQ9 scale; according to it, 381 (69.3%) participants were having mild depression and 60 (10.9%) had moderate depression. Figure 2 shows the depression score of participants.

Chi-square analysis was carried out to check the association between BMI and depression among study participants. The analysis showed an insignificant p-value, i.e. 0.135, which highlighted that there is no association between BMI and depression score in females of age 11-18. Table 2 depicts the chi-square analysis of BMI and depression.

**Table 2:** Chi-square analysis of study participants

BMI	Depression					p-Value
	None	Mild	Moderate	Moderate to severe	Severe	0.135
Underweight	16.5%	50.9%	8.4%	0.0%	0.0%	
Normal	2.9%	18.2%	2.5%	0.2%	0.0%	
Overweight	0.0%	0.2%	0.0%	0.0%	0.0%	
Obese	0.0%	0.0%	0.0%	0.0%	0.0%	

## Discussion

Obesity and depression are both prevalent during adolescence, and obesity may have been a trigger for adolescent depression; however, there is inconsistent evidence concerning how they interact over time [13]. Adolescence is a high-risk period for the development of such comorbidity in girls, with the nature of the risk changing over time. Early teenage depression is linked to an increased risk of later-onset obesity, whereas obesity, particularly in late adolescence, is attributed to an increased risk of later depression [14].

Our findings backed up and elaborated on the link between obesity and the onset of depression, particularly among women with the assistance of depression severity through PHQ9. The primary care assessment of mental disorders (PRIME-MD) is a great approach developed a decade ago to help primary care doctors make criteria-based diagnoses of five types of Diagnostic and Statistical Manual of Mental Disorders, DSM-IV disorders that are typically seen in medical patients: mood, anxiety, somatoform, alcohol, and eating disorders [15]. The PHQ is a three-page self-managed and administered variant of the PRIME-MD that has been thoroughly validated in two large studies that comprised 3,000 patients in eight primary care clinics and 3,000 patients in research settings [16]. The PHQ is presently the most widely used version in both clinical and research settings since it is completely self-administered and has diagnostic validity similar to the clinician-administered PRIME-MD. The PHQ9 is thus a dual-purpose questionnaire that may create provisional depressive disorder diagnoses as well as grade depression symptom severity using the same nine items [17].

However, as the PHQ9 becomes more widely used as a continuous measure of depression severity, knowing the likelihood of a severe or subthreshold depressive disorder at various cut points will be useful in our study. Obesity-to-depression and depression-to-obesity pathways have both found empirical support; a recent review found that 80% of community-based longitudinal studies examining obesity-to-depression pathways found evidence for statistical significance, while 53% of those examining depression-to-obesity pathways found evidence for statistical significance [18]. Parallel to our findings where (69.3%) participants were having mild depression and 60 (10.9%) had moderate depression, Shaffer et al. reported that the occurrence of depressive symptoms among children and teenagers aged 9-17 years is estimated to be 5%, while Teplin LA et al. stated obesity and overweight are common in teenagers, with over 30% of them being obese [19].

Despite efforts to reduce the escalating epidemic of childhood obesity, rates continue to rise, as does the social stigma that obese children undergo [20]. Obese adolescents have a higher frequency of scholastic and mental health difficulties than normal-weight adolescents, including poor academic performance and self-esteem, stress, psychological disorders, and a larger number of reported attempted suicides. Although there was no significant association of BMI with depression among our study participants, a similar study conducted in Sweden reported that after controlling for Nordic ancestry, neuropsychiatric diseases, family history of anxiety/depression, and socioeconomic level, obesity remained a significant risk factor for anxiety and depression in children and adolescents of school-going age. When compared to females in the general school population, obese girls had a 43% higher risk of anxiety and depression [21].

To recapitulate the social demographic factors from our study which were mostly categorized as "don’t know" indicated a lack of awareness while research suggested that various parent and family risk factors are linked to adolescent depression. They cover a wide range of topics, including parental cognitions, pathology, warm and emotional parenting techniques, individual coping with the family context, and family conflict. The findings strongly suggest that mothers and fathers are vital to include when contemplating youth depression risk and therapy for several reasons. More research has been focused in the last decade on the significant relationship between socioeconomic position and teenage depressive symptoms, which can be linked to demography, maternal parenting, and adolescent sense of purpose [22,23]. Female students with a low SES face numerous stressful life conditions and are more likely to acquire mental health problems than their counterparts with a better SES [23]. Furthermore, low SES is associated with a greater burden in several aspects of daily life as well as greater exposure to stressful life situations, but this effect was comparably minor in our study because the majority of students came from the middle SES [24].

## Conclusions

The BMI score of study participants seemed to be underweight or normal. The study could not rule out the association of BMI with depression. However, according to the PHQ9 scale score, many participants were screened as sufferers of mild to moderate depression which is alarming, as depression at the age of 11-18 years may predispose young girls to chronic disease and other psychological conditions. The current study only screened the prevalence of depression among young girls; however, diagnosis of depression based on clinical symptoms and its prompt treatment is recommended. Furthermore, the association of BMI with depression must be evaluated in girls with increased BMI.
